# A Narrative Review of the Launch and the Deployment of Telemedicine in Italy during the COVID-19 Pandemic

**DOI:** 10.3390/healthcare10030415

**Published:** 2022-02-23

**Authors:** Daniele Giansanti, Giovanni Morone, Alice Loreti, Marco Germanotta, Irene Aprile

**Affiliations:** 1 Centro Nazionale Tecnologie Innovative in Sanità Pubblica, Istituto Superiore di Sanità, 00161 Rome, Italy; 2 Dipartimento di Medicina Clinica, Sanità Pubblica, Scienze della Vita e dell’Ambiente, Università degli Studi dell’Aquila, 67100 L’Aquila, Italy; giovanni.morone@univaq.it; 3 Facoltà di Medicina e Psicologia, Università Sapienza, 00185 Roma, Italy; alicettaloreti@hotmail.com; 4 Istituto di Ricovero e Cura a Carattere Scientifico Fondazione Don Carlo Gnocchi, 50143 Florence, Italy; mgermanotta@dongnocchi.it (M.G.); iaprile@dongnocchi.it (I.A.)

**Keywords:** COVID-19, telemedicine, digital health, eHealth, mHealth, telehealth, telemonitoring, telerehabilitation

## Abstract

Telemedicine is making an important contribution to the fight against the COVID-19 pandemic and to supporting the health domain. Its use has registered initial problems with often-patchy practise. The objective of this study was to analyze the launch and deployment of telemedicine in Italy through a narrative review. The narrative review faced two points of view: (a) the first point of view revised the institutional initiatives of the Italian government developed to promote the use of telemedicine; (b) the second point of view reviewed the evolution of scientific literature in the sector, with reference to the Italian situation. In the second point of view, we applied both a standard narrative checklist and an eligibility approach. The first point of view reported an analysis of national documents aimed at promoting, through indications and recommendations, the use of telemedicine. The second point of view analyzed 39 qualified references. The analysis highlighted: (a) that initially, there was a disorientation, followed by reflections that emerged immediately after; (b) a telemedicine application not only in the traditional sectors (e.g., diabetology, cardiology, oncology, neurology) but also in new and fields never explored before; and (c) a high level of acceptance and a desire to continue in the after-pandemic future (which emerged in some studies through dedicated questionnaires). The study offers stimuli for both stakeholders and scholars to improve the use of telemedicine during the pandemic and in the future.

## 1. Introduction

Telemedicine as a diagnostic, monitoring, and rehabilitation treatment tool is showing great potential during the pandemic, as highlighted by Bahsnur et al. [1]. We believe that the current spread of telemedicine, compared with previous pandemic situations, can be explained by the simultaneous occurrence of unprecedented conditions of technological availability and exceptional medical circumstances. In fact, one aspect that drove this boom was the vastness of the pandemic, the most terrible of the past century; however, the real engine of the boom of telemedicine during the epidemic for the SARS-CoV-2 virus of 2019, has been mobile technology based on smartphones [2]; these are capable of hosting telemedicine applications, for example, based on wearable sensors, which in the past needed proper technological solutions [3]. During each wave of the pandemic’s evolution, telemedicine has shown exponential development [2]. In reference to the Italian situation, despite the available innovative solutions, cultural barriers and organization limits did not allow an easy introduction of these solutions to the health domain. The introduction happened with lights and shadows [4]. We can identify two periods of maturation of the use of telemedicine during the pandemic. During the first period, around the first lockdown (March–July 2020), two critical issues were found. The first issue can be summarized as a lost opportunity to widely spread telemedicine for long-term patients, as highlighted by Omboni [5]. The author believes that Italy was unprepared to use telemedicine in the first phase of the emergency. The second issue can be summarized as a lost opportunity to provide adequate telemedicine services to subjects renouncing the emergency recovery. Vigano et al. [6], highlighted that (a) in the first phase of the pandemic there was a significant decline in the number of patients who accessed the emergency room, and hospitalized patients; (b) this would have caused a presumable increase in health needs in the immediate future, to possibly be addressed with remote techniques, which were not completely usable at the time. After the first lockdown, a period of maturation can be identified, in which responses to some critical issues began to appear; these were based exceptional needs related to the pandemic that emerged, and several institutional indications on the use of telemedicine and in the area of interventions were produced. In particular, numerous reports by the Istituto Superiore di Sanità (the Italian National Institute of Health), focused both on telemedicine and on sectors needing telemedicine, have been published (such as, for example, the reports in [7,8,9,10]). Other stakeholders, such as the Regional Government and the Italian Ministry of Health, have also activated initiatives to promote the use of telemedicine. We can report as political initiatives:

- The drafting of national guidelines [11] for the provision of telemedicine services by the Ministry of Health, to avoid the patchy use of telemedicine and make telemedicine health services officially recognized health services that will have the same value as those in existence. It is understood that the doctor will always decide whether to use them or not.
- Regional initiatives aimed at the standardization and homologation of telemedicine services such as the one of the Lazio region [12], as a non-exhaustive example.

## 2. Purpose of the Study

The purpose of the study was to address the launch and deployment of telemedicine in Italy through a narrative review, addressing both the promotion initiatives at national level and the experiences of application also at the level of acceptance. All of this facilitates both answering the important question of “how telemedicine started in Italy during the pandemic and how it is going”, and making a point about the use of telemedicine in Italy.

## 3. Methods

This narrative review faced two points of view: (a) the first was aimed at analysing institutional government initiatives designed to promote the use of telemedicine and to raise awareness among stakeholders; (b) the second was aimed at analysing the evolution of scientific literature in the sector, through a Pubmed overview, with reference to the Italian situation. We followed both a narrative checklist and an eligibility approach, based on a scoring system (with different parameters and a score with five levels) applied by two qualified experts, to include each reference found in the second point of view.

We followed the narrative checklist reported in [13].

The manuscript was developed in accordance with this checklist, which requires compliance with some qualifying points in the development of the document, starting from the title and ending with the conclusions.

Table 1 shows the parameters used for the qualification process applied, before inclusion, on the references found in the second point of view.

We assigned a score to these parameters, ranging from a minimum score of one (very poor) up to a maximum of five (outstanding). As far as the “added contribution to the field” parameter is concerned, we used weighing. 

Both to consider the criticality of the first phases of the pandemic and to relativize the reference to the importance of the first period, the assigned vote was multiplied by:

- A factor × 1.3 (for studies published in the first three months of the pandemic).
- A factor × 1.15 (for studies published in the period ranging from 1 to 6 months of the pandemic).

The study was excluded if, regardless of the score, there were critical issues of conflict of interest (for example, if it was conducted without guarantees of objectivity by the system manufacturer). The reference was included in the review if all parameters after weighing showed a score higher than three in AND logic. 

The keys reported in Table 2 were applied in the second point of view.

## 4. Results

### 4.1. An Overview of Italian National Recommendations and Indications

In Italy, an important role in fighting the pandemic was played by the Istituto Superiore di Sanità (ISS). The ISS defined various working groups [14] with ISS researchers and experts on the various strategic issues related to the fight against the pandemic. The groups also worked in synergy with each other. Important products from the working groups were the ISS COVID-19 Reports containing guidelines and recommendations for all the insiders in the health domain. The COVID-19 Reports provide essential and urgent information for emergency management and are subject to updates. These reports were produced in the national language] and translated into English or other languages [15] to share/export the knowledge. We accessed the online archive and focused on content dedicated to telemedicine. Table 3 shows the COVID-19 reports that dealt directly or indirectly with telemedicine.

The first document [7] provided support for the realization of services in Telemedicine during a COVID-19 emergency, offering indications, identifying operational problems, and proposing solutions supported by evidence, but that are also easily dispensable in practice. The indications aimed to be used in various combinations to provide health services and psychological support; they also aimed to proactively monitor the health conditions of people in quarantine, in isolation, after discharge from the hospital, or of those who were isolated at home due to the rules of social distancing but were in need of continuity of care, even if they were not COVID-19 infected. The second document [8] provided the scientific indications to support the implementation of telemedicine health services for pediatric patients, both in early childhood and in developmental age, and during the different phases of the COVID-19 pandemic. It described how telemedicine can solve operational problems in managing the doctor–patient–family relationship in the pediatric field. It also provided concrete elements for the definition of specific characteristics, and the eligibility and exclusion criteria of the pediatric patient, also affected by rare or common chronic diseases.

The third document [9] illustrated the results of the development and submission of a survey (in September 2020) proposed by the National Centre for Innovative Technologies in Public Health and the National Centre for Rare Diseases of the ISS; its aim was to investigate the state of use of technologies (also based on telemedicine) by people with frailty, disabilities, and rare diseases. The document was intended to report evidence to stakeholders through the survey tool which played a sensor role. The fourth document [10] reported guidelines for the appropriate support of children with adrenal insufficiency during the current SARSCoV-2 pandemic emergency. Contact with reference centers to ensure advice from specialists was highly recommended, also using telemedicine systems.

All four of the documents [7,8,9,10] also highlighted particular attention to rare diseases and the frailty towards which telemedicine can play an important supporting role.

### 4.2. An Overview of Italian National Scientific Literature Production

The eligibility process applied to the selected references, after the elimination of the duplicated ones, returned 39 works [5,16,17,18,19,20,21,22,23,24,25,26,27,28,29,30,31,32,33,34,35,36,37,38,39,40,41,42,43,44,45,46,47,48,49,50,51,52,53], including a review focused on the relationship between telemedicine and radiotherapy [39]. 

A total of 17 studies were published in 2021, and the remaining 22 in 2020. 

The eligibility process also showed that the selected papers did not show critical issues regarding conflict of interest.

**Figure 1:**
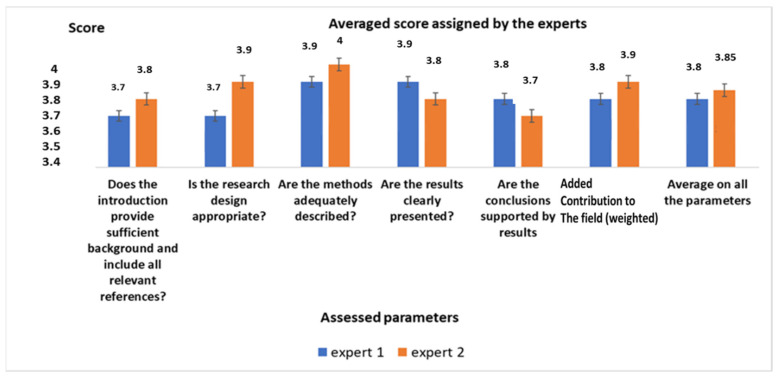
Output from the qualification process.

Figure 1 reports the average scores assigned by the two experts after the weighing process, both for each parameter and averaged for all the parameters.

Table 4 reports the references of the studies selected in the narrative review using the eligibility process with a summary of their focus. 

The studies show:

- An initial disorientation [5,33,51] in the use of telemedicine, during the implementation period of the guidelines/recommendations [7,8,9,10] and before the standardization initiatives [11,12];
- The scientific reflections regarding telemedicine use [46] following the immediate period after the first Italian national lock-down;
- The differentiated applications;
- An expansion of the telemedicine boundaries;
- A high acceptance, as tested through specific questionnaires in some studies.

#### 4.2.1. Disorientation in Telemedicine Applications Emerging in Some Studies

The study reported in [5] expressed dissatisfaction with the lost opportunity to widely spread telemedicine during the lockdown, wherein it is reported, and quoted verbatim: “Italy was found unprepared to manage lockdown patients with chronic diseases, due to limited availability and diffusion of large-scale telemedicine solutions.” Among the specific causes hindering the implementation of effective telemedicine solutions, the author indicates, specifically for long-term patients management: (a) the scattered distribution and heterogeneity of available tools; (b) the lack of integration with the electronic health record of the national health system; (c) the poor interconnection between telemedicine services operating at different levels; (d) the lack of a real multidisciplinary approach to the patient management; and (e) the heavy privacy regulations and lack of clear guidelines, together with the lack of reimbursement. 

In addition, the study reported in [51] emphasized that Italy did not include telemedicine in the essential levels of care granted to all citizens within the National Health Service, while other nations authorized, reimbursed, and actively promoted the use of telemedicine. The study stimulated the stakeholders to take action in the direction of telemedicine. The study in [33] discussed the legal problems in telemedicine delivery, ranging from profiles on the subject of authorization and accreditation to those concerning the protection of patient confidentiality.

#### 4.2.2. Reflections Emerging after the Italian National Lockdown

The study in [46] highlighted that: (a) the Italian lockdown model (in March–May 2020) has been imitated by many other states; (b) Italy was probably not a model in the use of telemedicine. However, there was an opportunity to reflect on this and inspire models that could be useful after the first lock down period. The following sectors on which to focus during the pandemic were detected [46]:

- Telemedicine and fragility for multiple chronic diseases;
- Certainly, a very important sector where telemedicine must intervene is that of the frail: those subjects suffering from single or multiple chronic pathologies (often elderly, but not always), the frequently disabled, and those with an unstable health status are particularly vulnerable in the case of COVID-19 infection;
- Telemedicine and fragility for rare diseases;
- As is well known, a rare disease can generate multiple chronicity and disabilities together. A telemedicine application must, in this case, be tailored to the patient;
- Television, telecooperation, and teleconsultation;
- Traditional telemedicine means that during the pandemic, social distancing and the minimization of the risk of contagion were possible;
- The expansion of telemedicine boundaries;
- The expansion to new applications could be possible due to the pandemic;
- New models for pulmonary rehabilitation using telemedicine;
- A patient returning home after weeks of intubation needed a properly designed home rehabilitation program, also based on pulmonary stimulation tools suitably integrated into telemedicine.

#### 4.2.3. Collected Evidence of Telemedicine Use

By analyzing the publications found in Pubmed to date, we can trace a picture of telemedicine use in the health domain regarding monitoring, surveillance, and continuity of care. 

We find various applications of telemedicine in diabetology [17,22,23,27,41] and also in children [31], where we see the use of ICT integration tools with self-assessment devices. Cardiology has also recorded the use of telemedicine. The study in [48] described a telemedicine experience in heart failure management during COVID-19. The study in [36] analyzed data from three telemedicine centers focusing on heart care. The study in [40] reported the evolution of a multidisciplinary center for myocarditis towards a telemedicine system. Important applications are recorded in oncology, such as in breast cancer [21], lung cancer [25], connections to the stoma centers [29], the management of patients with differentiated thyroid cancer [39], and related radiotherapy applications [35]. The neurology sector has also recorded an important use of applications in Parkinson’s disease [44]; in the use of telemedicine for the multidisciplinary assessment of patients with Amyotrophic Lateral Sclerosis [47]; for multiple sclerosis [28]; for a headache specialist center; and in applications of telephysiotherapy [47]. The overview also reported the application of telemedicine in other sectors less common in telemedicine solutions, such as: colorectal surgery [24]; wound care [20]; urological benign diseases [32]; endocrinology [34]; inflammatory bowel disease [37]; teleconsulting with a bariatric center; and chronic liver disease [42]. There has been the use of systems, in some simple cases, such as telephony [32,50], and in other more complex cases, such as specialized servers [53], which have allowed the application of telemedicine with success. 

#### 4.2.4. Example of the Expansion of the Boundaries

The boundaries of the use of telemedicine have been expanded during the pandemic. Three examples are show in transgender mental health monitoring [26], in the field of the abortion [18,19], and in the field of the animal-assisted therapy. Transgender people are a vulnerable group with a higher incidence of mental health issues and, during the COVID-19 outbreak, they may have faced psychological, physical, and social obstacles. The study in [26] evaluated the impact of the pandemic and access to health care services during the COVID-19 pandemic on the mental health of transgender people living in Italy. An anonymous web-based survey was conducted among transgender people living in Italy. It highlighted how telemedicine services may serve to mitigate negative psychological effects. The studies in [18,19] focused on the application of telemedicine in the field of abortion. Induced abortion is legal in Italy, but with restrictions. The online abortion provider Women on Web serves as an alternative way to access abortion. The study highlighted an increase in requests during the COVID-19 pandemic compared with the previous years without a pandemic (when its use was not sensibly appreciable). The most common reasons for requesting a telemedicine abortion through the system were privacy-related; however, this shifted to COVID-19-specific reasons during the pandemic. Another example of the expansion of the boundaries of telemedicine in complementary and alternative medicine is reported in [54]. The latter is a survey that was administered remotely to quantify the impact of animal-assisted therapy during the lock down. Through the survey, which also reported as a self-assessment test for anxiety, it was shown that pet owners had lower levels of anxiety.

#### 4.2.5. Examples on the Acceptance of Use

Several studies have accompanied the use of telemedicine with the application of questionnaires (in some cases even standardized) to investigate acceptance and satisfaction [21,22,25,29,38,43,47], from which the desire to continue with telemedicine even in post-pandemic periods have also clearly emerged, directly or indirectly. Telemedicine received a high degree of acceptance, for example, in oncology, where both a study on patients with breast cancer [21] and in the output of a project on lung cancer monitoring [25] displayed this. The study in [22] investigated the individual and contextual determinants of the perceived quality of telemedicine and teleassistance services, and willingness to continue with them among patients with diabetes. The study showed both a high level of acceptance and several determinants. These socio–demographic and correlated factors should be considered in the implementation of care pathways integrating in-person visits with the telemedicine. In addition, applications in neurology showed a high acceptance, as in the case of a study of the multidisciplinary assessment of patients with amyotrophic lateral sclerosis using telemedicine [47], and in a study embedding telephysiotherapy services [43]. Notably, the study in [38] also assessed the positive impact of a teleconsulting technology in a single bariatric center. Both interesting and innovative for oncology is the study in [29], which reported a high level of acceptance from patients involved in the experience of telemedicine consultations at a stoma center.

## 5. Discussion

The COVID-19 pandemic, as highlighted by Negrini et al. [4], represented an important engine for the development of telemedicine in Italy. Here, we have seen two important phases in the launch of the telemedicine. Many critical issues in the second phase have been addressed, and efforts have been made to improve the usability of telemedicine services, as well as standardization aspects, through institutional and political actions.

In this study we resumed these changes and reported an overview based on two points of view.

The first point of view reported the initiatives for issuing recommendations and indications for the use of telemedicine by the ISS through public reports [7,8,9,10]; these were in the national language and translated, in many cases, into English, and in other cases, into other languages. These reports have been a particular stimulus on the national scene for the use of broad-spectrum telemedicine, particularly in the case of various types of frailties, and also due to rare diseases.

The second point of view reported an analysis of the literature from Pubmed to examine the spread of telemedicine. This analysis highlights:

- An initial disappointment [5,33,51] in relation to the low use of telemedicine due to problems that are not only operational, but also bureaucratic and legislative.
- A subsequent broad-spectrum use in traditional applications—such as diabetology, cardiology, oncology, and neurology—but also in original sectors, such as application in bariatric centers, wound care, urological benign diseases, endocrinology, inflammatory bowel disease, and chronic liver disease (Table 3).
- New emerging applications, such as mental health in transgender people [26], telemedicine applied to abortion [18,19], and the assessment of the impact of the animal-assisted therapy [54].
- Studies based on surveys [21,22,25,29,38,43,47] that have shown a high acceptance of telemedicine, the determinants, and a direct or indirect interest in continuing with these solutions in the future.

If we compare the development of telemedicine during the pandemic in Italy with the USA, a nation that showed one of the best telemedical preparedness [1], we can highlight some important considerations. As we highlighted in [54] in a comment to [1], the telemedicine boom during the COVID-19 has not been identical across the world, for example it was different between the USA, Italy and Europe. Different regulations, and a less enlightened and more conservative political approach have, in many cases, hampered the spread of telemedicine in the first months of the pandemic. To cite a first example, whereas in the United States, the system based on medical insurance has clearly defined the reimbursement procedures, in Italy and in Europe this has not happened so explicitly. In USA, there were immediately derogations to the law for the use of messaging and video communication systems to be applied to telemedicine. Europe has not clearly made explicit derogations to current regulations in the first phases of the pandemic. However, after an initial disorientation, and some phasing initiatives, as shown in [46], telemedicine began to be used and stimulated, supported by a public- and equity-based healthcare approach. Then, in the USA—where the health system itself, based on private insurance, had allowed a rapid response to the use of telemedicine—scholars began to question, after a few months, the disparities and inequalities of telemedical treatment based on a private health system [55]. 

### Limitations

The study, based on a review of Pubmed and of the ISS online database, has limitations. As regards the publications, it analyzed scientific productions in the English language. It did not analyze publications in other languages (Spanish, French or Italian). It analyzed only the online database Pubmed, which, however, is strategic in the health domain, where the overview is focused. It is not a systematic review, given that the topic (the launch of a technology) required a type of investigation based on polyhedral sources (some non-scientific publications) and specific filtering more suitable for other types of reviews, such as narrative reviews, realistic reviews, hermeneutical reviews, rapid reviews, or simple overview reviews (all reviews admitted in the journal). 

## 6. Conclusions

In conclusion, the study highlights that: (a) in Italy, after the first moment of disorientation, telemedicine was used broadly and effectively; (b) new fields of telemedicine application were also explored; (c) dedicated questionnaires showed a high level of acceptance of telemedicine, and a desire to continue using this technology; (d) important suggestions emerged to invest in the use of telemedicine during the pandemic, and in the future after the pandemic.

By comparing the results of this study with other studies focused on other realities based on a different approach to the health system (for example, a private approach), we can highlight how the COVID-19 pandemic has been a stimulus for the development and use of telemedicine, and for the reviewing of regulations and policies in order to improve the use of this service, which can represent an instrument of equity and protection (thanks to social distancing) in this period, and an opportunity for the future.

## Figures and Tables

**Table 1:** Parameters used for the eligibility.

	Score (1 = min; 5 = max)	Weighting
Does the introduction provide sufficient background and include all relevant references?		N.A.
Is the research design appropriate?		N.A.
Are the methods adequately described?		N.A.
Are the results clearly presented?		N.A.
Are the conclusions supported by results?		N.A.
Added contribution to the field		*p* = 1.3 or 1.15 *

* 1.3 was used for the studies published in the first three months of the pandemic, and 1.15 was used for the studies published in the period ranging from 1 to 6 months of the pandemic.

**Table 2:** The keys applied in the search (COVID-19 was also changed with SARS-Cov-2 during the searches).

Applied Keys
*((telemedicine [Title/Abstract]) AND (COVID-19 [Title/Abstract])) AND (Italy [Title/Abstract])*
*((telehealth [Title/Abstract]) AND (COVID-19 [Title/Abstract])) AND (Italy [Title/Abstract])*
*((eHealth [Title/Abstract]) AND (COVID-19 [Title/Abstract])) AND (Italy [Title/Abstract])*
*((mHealth [Title/Abstract]) AND (COVID-19 [Title/Abstract])) AND (Italy [Title/Abstract])*
*((digital health [Title/Abstract]) AND (COVID-19 [Title/Abstract])) AND (Italy [Title/Abstract])*
*((telerehabilitation [Title/Abstract]) AND (COVID-19 [Title/Abstract])) AND (Italy [Title/Abstract])*
*((telemonitoring [Title/Abstract]) AND (COVID-19 [Title/Abstract])) AND (Italy [Title/Abstract])*
*((telemedicine [Title/Abstract]) AND (COVID-19 [Title/Abstract])) AND (Italy [Title/Abstract])*

**Table 3:** ISS reports dealing with the telemedicine.

Report	Cited Report	Brief Description of the Focus
12/20	Gabbrielli F et al. [7]. Interim provisions on telemedicine healthcare services during COVID-19 health emergency. Version from 13 April 2020.	Recommendations for telemedicine employment
60/20	Gabbrielli F et al. [8]. Interim guidance on Telemedicine health services for Paediatrics during and beyond COVID-19 pandemic. Version from 10 October 2020.	Recommendations for telemedicine employment in pediatrics
14/21	Giansanti D. et al. [9]. Technologies to support frailty, disability and rare diseases: development and submission of a survey during the pandemic emergency COVID-19. Version from 18 June 2021.	Outcome from a survey on the use of technologies (also telemedicine) during the pandemic
24/20	ISS [10]. COVID-19 Rare Diseases Working Group Interim guidelines for the appropriate support of children with adrenal insufficiency during the current SARSCoV-2 pandemic emergency. Version from 10 May 2020.	Interim guidelines for the appropriate support of children with adrenal insufficiency also using telemedicine

**Table 4:** Cited articles with a summary of the focus.

Cited Article	Brief Description of the Focus
Caponnetto, V., et al. [16]. The COVID-19 Pandemic as an Opportunity to Improve Health Care Through a Nurse-Coordinated Multidisciplinary Model in a Headache Specialist Center: The Implementation of a Telemedicine Protocol.	The contribution described the implementation of a structured telemedicine protocol during the COVID-19 pandemic. The study performed a quality improvement study in a Headache Specialist Center. A total of 207 telemedicine visits involving 100 patients was performed. Telemedicine-facilitated follow-ups, ensuring multidisciplinary care and high patient satisfaction, justifying its wider adoption in headache care.
Lazzeroni, P. et al. [17]. Improvement in glycaemic control in paediatric and young adult type 1 diabetes.	The aim of the work was to assess metabolic control before and after lockdown in the cohort of type 1 diabetes patients, followed-up by telemedicine. A total of 139 patients were enrolled. Results showed a global improvement in mean HbA1c, with a stronger result for patients with a previous non-satisfactory control. No worsening of metabolic control was shown for patients.
van Ooijen, L.T., et al. [18]. A trans-national examination of the impact of the COVID-19 pandemic on abortion requests through a telemedicine service.	This contribution is logically connected to the next, giving a transnational overview of the topic.
Brandell, et al. [19]. Telemedicine as an alternative way to access abortion in Italy and characteristics of requests during the COVID-19 pandemic.	Induced abortion is legal in Italy, but with restrictions. The online abortion provider Women on Web serves as an alternative way to access abortion. The study highlighted an increase in requests during the COVID-19 pandemic compared with the previous year (12% in the first 9 months). The most common reasons for requesting a telemedicine abortion through WoW were privacy-related (40.9%); however, this shifted to COVID-19-specific (50.3%) reasons during the pandemic.
Scalise, A., et al. [20]. What COVID-19 taught us: New opportunities and pathways from telemedicine and novel antiseptics in wound healing.	The aim of this multidisciplinary work was to highlight the importance of a new pathway of wound care with a patient-based therapeutic approach, tailored treatments based on the characteristics of the wound, and fast tracks focused on outpatient management, reserving hospital assessment only for patients with complicated or complex wounds.
Bizot, A., et al. [21]. Multicenter evaluation of breast cancer patients’ satisfaction and experience with oncology telemedicine visits during the COVID-19 pandemic.	The study examined the satisfaction of 1299 patients with breast cancer who underwent teleconsultations during this period. Standardized questionnaires were electronically proposed. Patients were satisfied with oncology teleconsultations during the COVID-19 pandemic. Teleconsultation may be an acceptable alternative follow-up modality in specific circumstances.
Maietti, E., et al. [22]. The experience of patients with diabetes with the use of telemedicine and teleassistance services during the COVID-19 pandemic in Italy: Factors associated with perceived quality and willingness to continue.	The purpose of this study was to investigate the individual and contextual determinants of the perceived quality of the telemedicine and teleassistance services, and the willingness to continue with them, among patients with diabetes. The study identified several determinants of perceived quality and willingness to continue. These socio–demographic and related factors should be considered in the implementation of care pathways integrating in-person visits with telemedicine.
Tornese, et al. [23]. The effect of the COVID-19 pandemic on telemedicine in pediatric diabetes centers in Italy: Results from a longitudinal survey.	The study investigated the increase in the use of telemedicine in two diabetes centers during the evolution of the pandemic. Eighty-two percent of responder centers reported an increase in the use of telemedicine, with tele visits by video calling implemented in over half of the centers. There was a significant increase in the number of centers formally tracking telemedicine use and obtaining reimbursement from the national health service (42% vs. 29% and 62% vs. 32%; *p* < 0.001, respectively). No reimbursement was provided to centers not using televisits. The study highlighted that telemedicine from a procedure with a lack of traceability has become a new structured reality that may help our pediatric patients beyond this pandemic.
Gallo, G., et al. [24]. Telemedicine in Colorectal Surgery Italian Working Group, Grossi U. E-consensus on telemedicine in colorectal surgery: a RAND/UCLA-modified study.	The aim of the study was to reach consensus among experts on the possible applications of telemedicine in colorectal surgery. A panel of experts was defined. The panel voted against the use of telemedicine for a first consultation. Consensus was achieved in all but one statement concerning the cost of a teleconsultation. There was strong agreement on the usefulness of teleconsultation during the follow-up of patients with diverticular disease after an in-person visit.
Pardolesi, A., et al. [25]. Telemedicine for management of patients with lung cancer during COVID-19 in an Italian cancer institute: SmartDoc Project.	The study reported the outcome of a project on lung cancer monitoring. A total of 83 patients participated in the SmartDoc project and received a teleconsultation. A survey was proposed to the participants. A “complete satisfaction” score (5 out of 5 points) was reported in 70.59% of all the respondents; most patients (76.5%) preferred video-consulting and defined it as better than or comparable to an in-person visit.
Gava, G., et al. [26]. Mental Health and Endocrine Telemedicine Consultations in Transgender Subjects During the COVID-19 Outbreak in Italy: A Cross-Sectional Web-Based Survey.	The study evaluated the impact of the pandemic and the access to health care services during the COVID-19 pandemic on the mental health of transgender people living in Italy. An anonymous web-based survey was conducted among transgender people living in Italy. It highlighted how telemedicine services may serve to mitigate negative psychological effects.
Luzi, L., et al. [27]. Telemedicine and urban diabetes during COVID-19 pandemic in Milano, Italy during lock-down: epidemiological and sociodemographic picture.	A pilot study was conducted to assess the feasibility and efficacy of telemonitoring of glucose control in a cohort of diabetic patients. The study demonstrated a reduction in glycated hemoglobin at 3 months follow-up during the lock-down period, indicating glucose monitoring and remote control as a potential methodology for diabetes management.
Corea., et al. [28]. Telemedicine during the Coronavirus Disease (COVID-19) Pandemic: A Multiple Sclerosis (MS) Outpatients Service Perspective.	Televisits during the COVID-19 outbreak demonstrated their utility as a care delivery method for multiple sclerosis. Hence, it is vital to facilitate the implementation of this technology in common practice to both face infectious threats and increase accessibility to the health care system.
Dinuzzi, V.P., et al. [29]. Telemedicine in Patients With an Ostomy During the COVID-19 Pandemic: A Retrospective Observational Study.	During the lockdown period, 181 in-person and 99 telemedicine consultations were provided by a stoma center. A questionnaire was used to assess the acceptance. Of the 65 patients who completed the questionnaire, 82% indicated being extremely satisfied. The reorganization of stoma care services, including the availability of telemedicine, did not result in a decrease in the number of consultations provided. The results suggest that stoma care services using telemedicine may provide valid support for patients with an ostomy in the future.
Miceli, L., et al. [30]. Doctor@Home: Through a Telemedicine Co-production and Co-learning Journey.	The National Cancer Institute of Aviano, Italy, has recently launched a program called “Doctor @ Home” (D@H). The pillars of the program were described in the contribution.
Predieri, B., et al. [31]. Control Improvement in Italian Children and Adolescents With Type 1 Diabetes Followed Through Telemedicine During Lockdown due to the COVID-19 Pandemic.	Sixty-two children and adolescents with type 1 diabetes were enrolled in a study. Overall, in the children and adolescents, control improved during lockdown. Despite patients being confined to their homes and limited to exercise, the data suggest that the use of real-time measurement of glucose, continuous parental management, and telemedicine can result in beneficial effects.
Checcucci, E., et al. [32]. Uro-technology and SoMe Working Group of the Young Academic Urologists Working Party of the European Association of Urology. Implementing telemedicine for the management of benign urologic conditions: a single centre experience in Italy.	The use of telemedicine with phone-call visits, as a practical tool to follow-up with patients affected by urological benign diseases, was investigated on 607 patients. Telemedicine was shown to limit the number of instances of unnecessary access to medical facilities, and represented an important tool for the limitation of the risk of transmission of infectious diseases, such as COVID-19.
Ferorelli, D., et al. [33]. Medical Legal Aspects of Telemedicine in Italy: Application Fields, Professional Liability and Focus on Care Services During the COVID-19 Health Emergency.	The paper discussed of the legal problems on the telemedicine delivery ranging, from the profiles on the subject of authorization and accreditation to those concerning the protection of patient confidentiality.
Ceccato, F., et al. [34]. Telemedicine versus face-to-face consultation in Endocrine Outpatients Clinic duringCOVID-19 outbreak: a single-center experience during the lockdown period.	The study aimed to assess the efficacy of the emergency plan to continue the follow-up of outpatients in tele-endocrinology The study showed a similar outcome both in young and aged patients with endocrine diseases.
Di Franco, R., et al. [35]. COVID-19 and radiotherapy: potential new strategies for patients’ management with hypofractionation and telemedicine.	Cancer patients are at higher risk of COVID-19 infection because of their immunosuppressive state caused by both the tumor itself and the anticancer therapy adopted. In this setting, the radiation therapy clinical decision-making process was partly reconsidered; thus, to reduce treatment duration and minimize infection risk during a pandemic, hypofractionated regimens were revised. This review aimed to point out the importance of hypofractionated radio therapy and telemedicine in cancer patient management in the COVID-19 era.
Molinari, G., et al. [36]. Impact of 2020 SARS-CoV-2 outbreak on telemedicine management of cardiovascular disease in Italy.	The study analyzed data from three telemedicine dispatch centers focused in heart care. Records from the time interval March 1 2020 and April 1 2020 were compared with the corresponding periods in 2019. The comparative analysis of data showed a significant reduction in telemedicine electrocardiogram transmission.
Zingone, F., et al. [37]. Perception of the COVID-19 Pandemic Among Patients With Inflammatory Bowel Disease in the Time of Telemedicine: Cross- Sectional Questionnaire Study.	The study, based on a survey, demonstrated that lockdown had a significant impact on the psychological aspects of patients with IBD and suggest the need to increase communication with patients with inflammatory bowel disease (e.g., through telemedicine) to ensure that patients receive adequate health care, correct information, and proper psychological support.
Runfola, M., et al. [38]. Telemedicine Implementation on a Bariatric Outpatient Clinic During COVID-19 Pandemic in Italy: an Unexpected Hill-Start.	This paper aimed to evaluate the impact of teleconsulting technology in a single bariatric center on 33 booked participants. A total of 19 (57.6%) participated in the telemedicine program. No significant differences were found between participants and non-participants in terms of age and gender ratio. A total of 52.6% completed a survey reporting levels of satisfaction ranging from high to very high.
Klain, M., et al. [39]. Management of differentiated thyroidcancer through nuclear medicine facilities during COVID-19 emergency: the telemedicine challenge.	Th study investigated whether a telemedicine service carried out during the COVID-19 pandemic impacted the management of patients with differentiated thyroid cancer. The number of outpatient visits performed during the pandemic (n = 445) and by in-ward access in the corresponding period of 2019 (n = 525) was comparable. The findings demonstrated the utility of telemedicine tools to avoid the potential negative impact of interruption or postponement of diagnostic and/or therapeutic procedures.
Peretto, G., et al. [40]. Telemedicine in myocarditis: Evolution of a mutidisciplinary “disease unit” at the time of COVID-19 pandemic.	More than 300 patients coming from the whole Country are currently followed up at a specialized multidisciplinary outpatient clinic. Following the pandemic outbreak of the SARS-CoV-2 infection in Italy, the authors presented how the multidisciplinary output clinic rapidly evolved to a “telemultidisciplinary output clinic”, via a dedicated multitasking digital health platform.
Longo, M., et al. [41]. Glycemic control in people with type 1 diabetes using a hybrid closed loop system and followed by telemedicine during the COVID-19 pandemic in Italy.	The study was aimed at evaluating the metrics of glycemic control in people with type 1 diabetes using the hybrid closed loop (HCL) system during the COVID-19 lockdown. Adults with type 1 diabetes using HCL showed a significant improvement in most of the metrics of glucose control during the COVID-19 lockdown.
Guarino, M., et al. [42]. Use of Telemedicine for Chronic Liver Disease at a Single Care Center During the COVID-19 Pandemic: Prospective Observational Study.	The aim of this study was to analyze the benefits of using telemedicine services for patients with chronic liver disease at a tertiary care center in Italy during the COVID-19-mandated lockdown. During the lockdown in Italy, almost 400 visits were conducted using telemedicine. It was shown to be a useful tool for following up patients with chronic liver disease and for reducing the impact of the COVID-19 pandemic.
Negrini, S., et al. [43]. Acceptability of Telemedicine to Substitute Outpatient Rehabilitation Services in the COVID-19 Emergency in Italy: An Observational Everyday Clinical-Life Study.	The study investigated the feasibility and acceptability of telemedicine as a substitute for outpatient services in emergency situations. Telemedicine services included teleconsultations and telephysiotherapy. Continuous quality improvement questionnaires were also evaluated. A total of 325 teleconsulations and 882 telephysiotherapy sessions were provided in 15 days. Patients’ satisfaction with telemedicine was very high (2.8 out of 3).
Cilia, R., et al. [44]. Telemedicine for parkinsonism: A two-step model based on the COVID-19 experience in Milan, Italy.	During the COVID-19 crisis, a telemedicine program for patients with parkinsonism was boosted in Milan, Italy. This two-step model integrated a telenursing forward triage followed by video-consultations by experienced neurologists.
Capozzo, R., et al. [45]. Telemedicine for Delivery of Care in Frontotemporal Lobar Degeneration During COVID-19 Pandemic: Results from Southern Italy ct.	The study evaluated the multidisciplinary assessment of patients with frontotemporal lobar dementia using telehealth during the COVID-19 pandemic. The study indicated that telemedicine is a valid tool to triage patients with frontotemporal lobar dementia to increase practice outreach and efficiency.
Giansanti, D., [46]. The Italian Fight Against the COVID-19 Pandemic in the Second Phase: The Renewed Opportunity of Telemedicine.	The letter discussed the importance of telemedicine after the lock down as a means of continuity of care, maintaining “social distancing”.
Capozzo, R., et al. [47]. Telemedicine is a useful tool to deliver care to patients with Amyotrophic Lateral Sclerosis during COVID-19 pandemic: results from Southern Italy.	The study evaluated the feasibility of the multidisciplinary assessment of patients with Amyotrophic Lateral Sclerosis using telemedicine during the emergency determined by the COVID-19 pandemic. In a successive survey, most of patients were satisfied with the neurological interview (85%), the possibility to interact directly with the clinician while at home (85%), and the reduction in economic and time costs because they avoided unnecessary travel to the clinic.
Salzano, A., et al. [48]. Heart failure management during the COVID-19 outbreak in Italy: a telemedicine experience from a heart failure university tertiary referral centre.	The letter described a telemedicine experience in heart failure management during COVID-19, showing on 103 patients that telemedicine, in most cases, allowed a clinical decision to be reached.
Siniscalchi, M., et al. [49]. COVID-19 pandemic perception in adults with celiac disease: an impulse to implement the use of telemedicine.	The authors aimed to evaluate the application perception of the use of a large-scale remote consultation approach—based on a Web surveyi—in 651 Celiac Disease patients who require a lifelong gluten-free diet as therapy. The remote tool allowed assessment of their psychological perceptions.
Tolone, S., et al. [50]. Telephonic triage before surgical ward admission and telemedicine during COVID-19 outbreak in Italy. Effective and easy procedures to reduce in-hospital positivity.	The comment described the telephonic triage before surgical ward admission and telemedicine during the COVID-19 outbreak in Italy. It described effective and easy procedures to reduce in-hospital positivity.
Omboni, S. [5]. Telemedicine During the COVID-19 in Italy: A Missed Opportunity?	The letter stated that Italy was found unprepared to manage lockdown patients with chronic diseases, due to limited availability and the diffusion of large-scale telemedicine solutions; it stated that the epidemic should help to promote better use and a larger integration of telemedicine services in the armamentarium of health care services.
Ohannessian, R., et al. [51]. A Global Telemedicine Implementation and Integration Within Health Systems to Fight the COVID-19 Pandemic: A Call to Action.	The contribution highlighted that Italy did not include telemedicine in the essential levels of care granted to all citizens within the National Health Service, while other nations authorized, reimbursed, and actively promoted the use of telemedicine. The authors highlighted the challenges remaining for the global use and integration of telemedicine into the public health response to COVID-19 and future outbreaks.
Sossai, P., et al. [52]. Telemedicine and the 2019 coronavirus (SARS-CoV-2).	The contribution reported the experience of telemedicine conducted by hepatologists in a tertiary-care Center for Liver Disease of a University Hospital in Northern Italy, for a 2-week period during the COVID-19 pandemic, on 138 patients. The study emphasized the usefulness of telemedicine for maintaining continuity of care among patients with autoimmune liver diseases during the pandemic.
Rigamonti, C., et al. [53]. Rates of Symptomatic SARS-CoV-2 Infection in Patients With Autoimmune Liver Diseases in Northern Italy: A Telemedicine Study.	The contribution reported a project that used an online platform between general practitioners and patients, in order to reduce moving infected individuals and to perform diagnosis and treatment early on.

## Data Availability

Not applicable.
